# Supplementary material for: SYP72 interacts with the mechanosensitive channel MSL8 to protect pollen from hypoosmotic shock during hydration
Source: Nat Commun. 2022 Jan 10;13:73. doi: 10.1038/s41467-021-27757-9 (PMC8748641; doi:10.1038/s41467-021-27757-9)
Supplement: Supplementary file 1 — Supplementary Information [file 41467_2021_27757_MOESM1_ESM.pdf]

SYP72 interacts with the mechanosensitive channel MSL8 to protect pollen from hypoosmotic shock during hydration

Zhou *et al.*

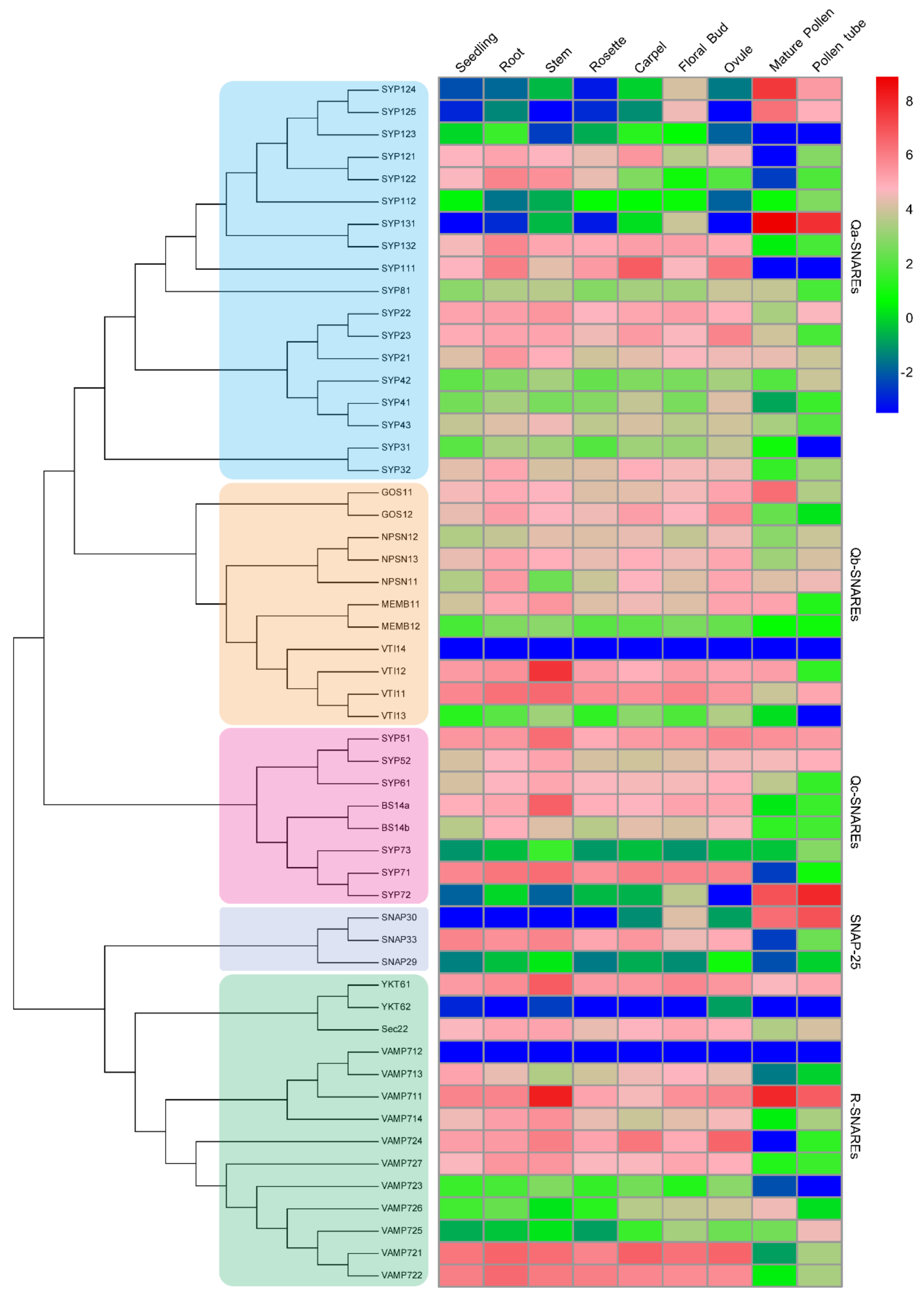

Supplementary Fig. 1 Expression pattern of *Arabidopsis SNARE* genes.

Left: Phylogenetic tree of 54 *Arabidopsis* SNAREs; Right: Heat map shows the expression of *SNARE genes* in both vegetative and reproductive organs. The expression level of each *SNARE gene* was expressed as log_2(FPKM+0.1). Publicly-available RNA-seq datasets including seedling (GSE32318), stem (GSE102694), root and rosette (GSE87760), carpel (GSE56326), floral bud (GSE45685), ovule (DRR044369), mature pollen (PRJNA194429), pollen tube (GSE98145) were used for expression level analysis. The phylogenetic tree was constructed with MEGA7 software using the Neighbor-Joining method. FPKM, fragments per kilobase of transcript per million mapped reads.

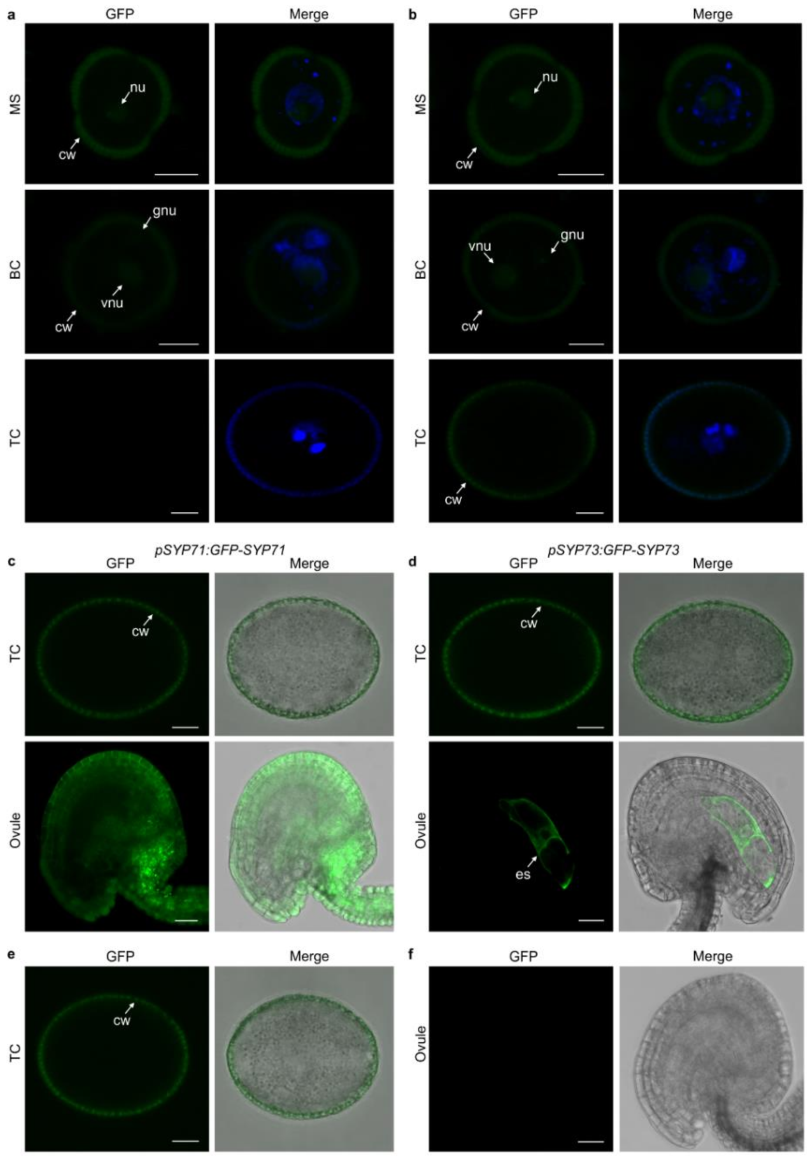

Supplementary Fig. 2 Expression pattern of three members of plant-specific *SYP7* family.

a, b Images of non-transgenic controls acquired using the same imaging conditions for *pSYP72:H2B-GFP* (**a**) and *pSYP72:GFP-SYP72* (**b**). GFP, GFP fluorescence; Merge, merged images for GFP and 4′,6-diamidino-2-phenylindole (DAPI) staining. Note: the nucleolus of microspore and bicellular pollen have weak auto-fluorescence. Auto-fluorescence was also observed in the pollen wall, but not in the ovules. **c, d** Analysis of *GFP-SYP71* (**c**) and *GFP-SYP73* (**d**) report lines revealed that *SYP71* and *SYP73* were detected in the female gametophyte, but not in the male gametophyte. Merge, merged images for GFP and DIC. **e, f** Images of non-transgenic controls for **c** and **d**. Note: auto-fluorescence was also observed in the pollen wall, but not in the ovules. MS, microspore; BC, bicellular pollen; TC, tricellular pollen; nu, nucleolus; gnu, nucleolus of the generative cell; vnu, nucleolus of the vegetative cell; cw, cell wall; es, embryo sac. Scaler bars, 5 μm for pollen grains and 20 μm for ovules. Observation of GFP in WT (**a, b, e, f**), *pSYP71: GFP-SYP71* (**c**) and *pSYP73: GFP-SYP73* (**d**) transgenic plants was repeated at least three times with similar results.

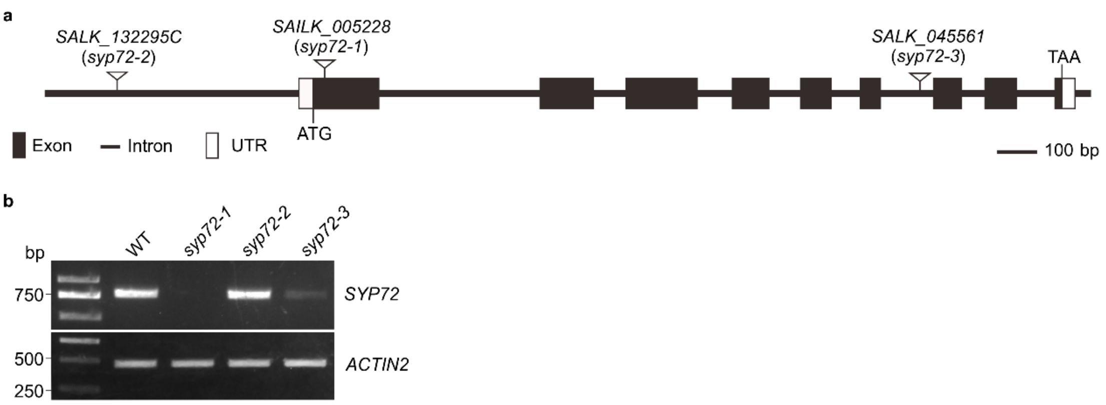

Supplementary Fig. 3 Identification of three T-DNA insertion lines in the *SYP72*. **a** Schematic diagram of the *SYP72* locus and locations of *syp72* T-DNA insertion sites. **b** The transcript levels of *SYP72* in three T-DNA insertion lines were determined by RT-PCR. *ACTIN2* was used as the internal control. RT-PCR experiments were performed independently three times with similar results.

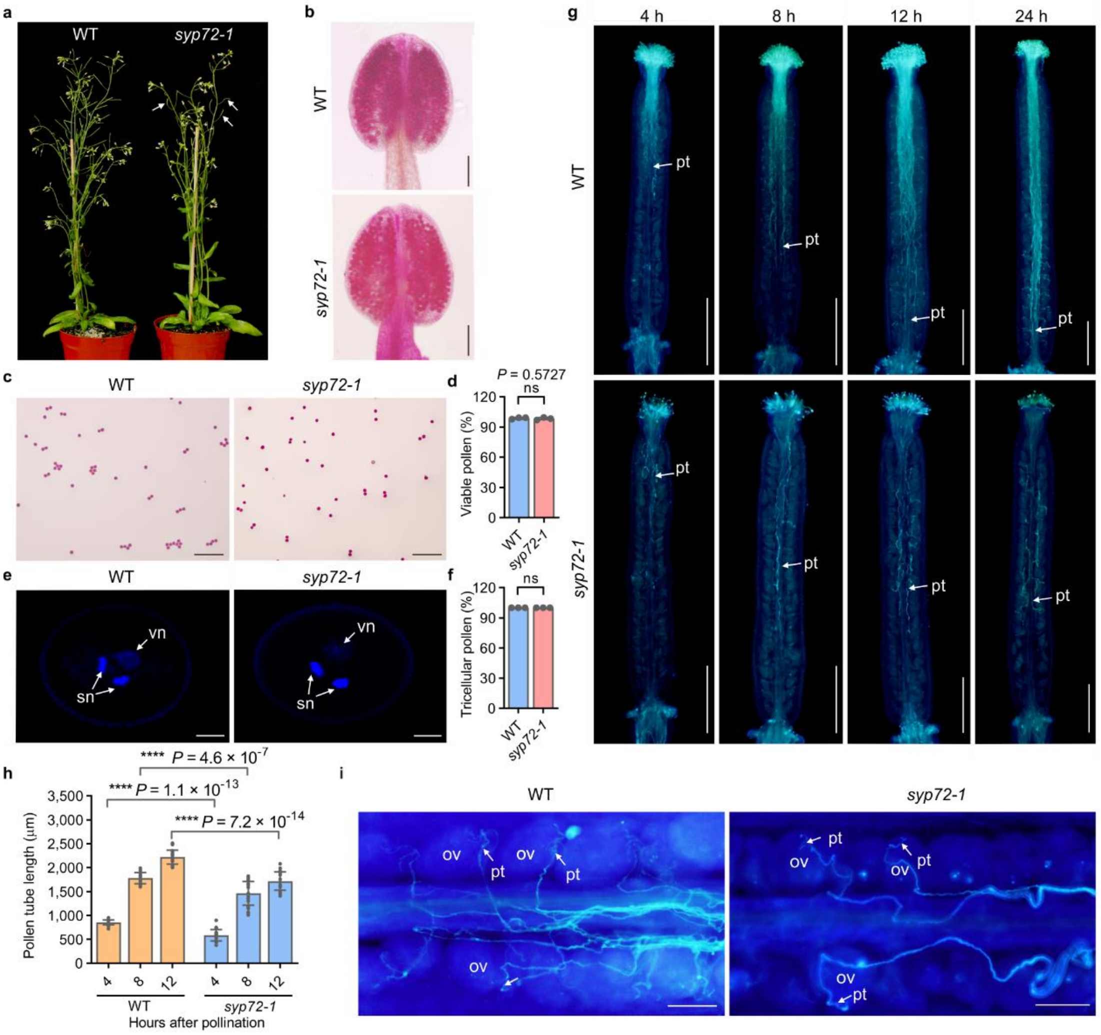

Supplementary Fig. 4 Phenotype characterization of *syp72* mutants.

a Representative images of WT and *syp72-1* plants. The arrows indicate *syp72-1* siliques containing aborted seeds. **b–d** Pollen viability from WT and *syp72-1* plants was determined by Alexander's staining. **b**, **c** Representative images of anther (**b**) and pollen grains (**c**) stained with Alexander's staining. Scale bars, 100 µm in (**b**) and 200 µm in (**c**). (n = 15 biologically independent anthers in **b**). **d** Percentage of viable pollen grains from WT and *syp72-1* plants. Data represent the mean ± SD from three independent experiments with scoring 500 pollen grains in each experiment. **e** Representative images of pollen grains stained with DAPI. Scale bars, 5 µm. **f** Percentage of tricellular pollen from WT and *syp72-1* plants. Each datum represents the mean from three independent experiments with scoring 30 pollen grains in each experiment. **g** Aniline blue staining assay of emasculated WT pistils pollinated after 4-24 hours pollination with WT or *syp72-1* pollen. Scale bars, 500 µm. sn, sperm cell nucleus; vn, vegetative nucleus; pt, pollen tube. **h** Statistical data of pollen tube length of the WT and the *syp72-1* mutant in WT pistil at different hours after pollination (HAP). Data represent the mean ± SD (n = 25 biologically independent pistils). **i** Representative images showing WT and *syp72-1* pollen tube targeting the WT ovules (n = 30 biologically independent pistils). Scale bars, 100 µm. Two-tailed Student's t-test was used for statistical analysis (ns, no significant difference, $P > 0.05$; ****$P < 0.0001$).

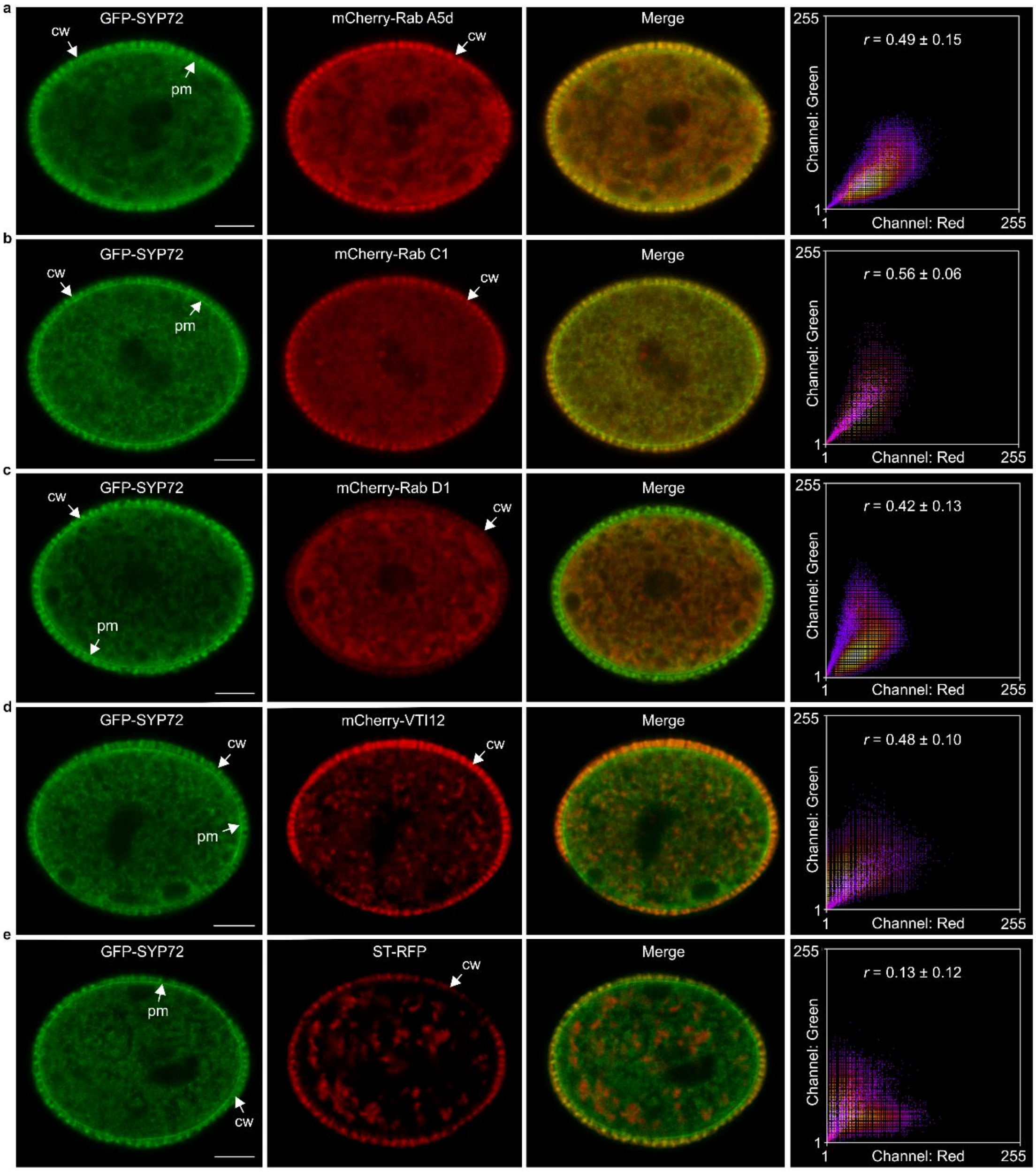

Supplementary Fig. 5 Subcellular location of SYP72 in *Arabidopsis* pollen.
a–e Confocal microscopy images showing the subcellular location of GFP-SYP72 and different known organelle markers including mCherry-Rab A5d (recycling endosome, **a**), mCherry-Rab C1 (post-Golgi, **b**), mCherry-Rab D1 (post-Golgi, **c**), mCherry-VTI12 (TGN/EE, **d**) and ST-RFP (Golgi, **e**) in pollen grains. Scale bars, 5 µm. The rightmost column shows the frequency scatterplot of merged images. Pearson correlation coefficients indicate the extent of colocalization between GFP-SYP72 and each known organelle marker. Data represent mean ± SD (n = 21 biologically independent pollen grains for mCherry-Rab A5d; n = 27 for mCherry-Rab C1; n = 27 for mCherry-Rab D1; n = 18 for mCherry-VTI12; n = 25 for ST-RFP). cw, cell wall; pm, plasma membrane.

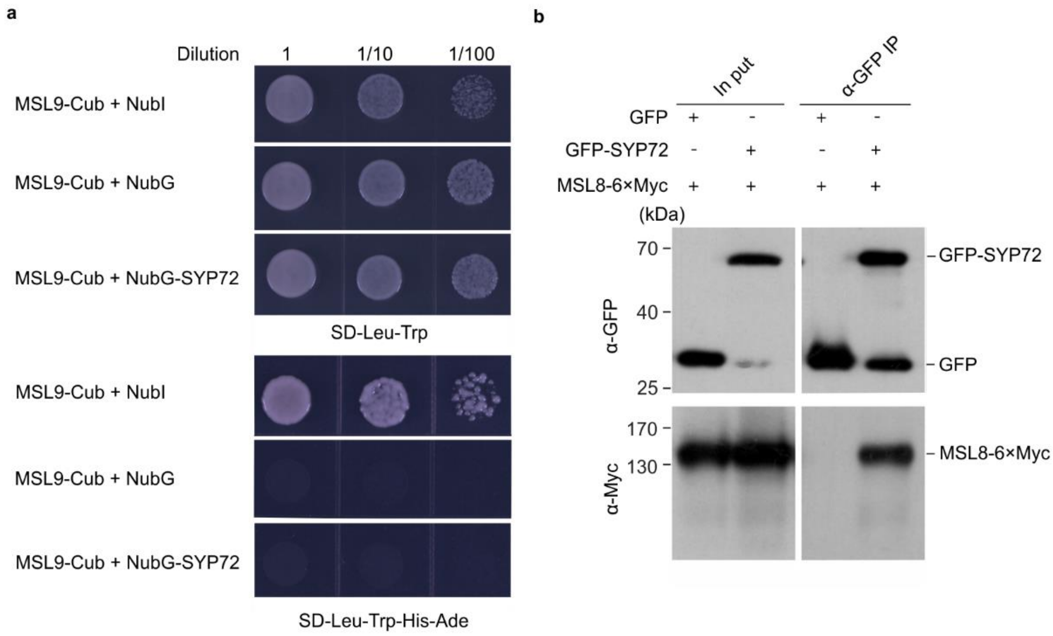

Supplementary Fig. 6 SYP72 interacts with the mechanosensitive channel MSL8, but not with the MSL9.

a Dual-membrane yeast two-hybrid assays with SYP72 and MSL9. **b** Co-IP assay of GFP-tagged SYP72 and Myc-tagged MSL8 in tobacco leaves. *35S:GFP-SYP72* and *35S:MSL8-6×Myc* were transiently coexpressed in the tobacco leaves. Tobacco leaves expressing *35S:GFP* and *35S:MSL8-6×Myc* were used as a negative control. Total proteins extracted from infiltrated leaves were used for Co-IP assay. Yeast two-hybrid and Co-IP experiments were repeated independently three times with similar results.

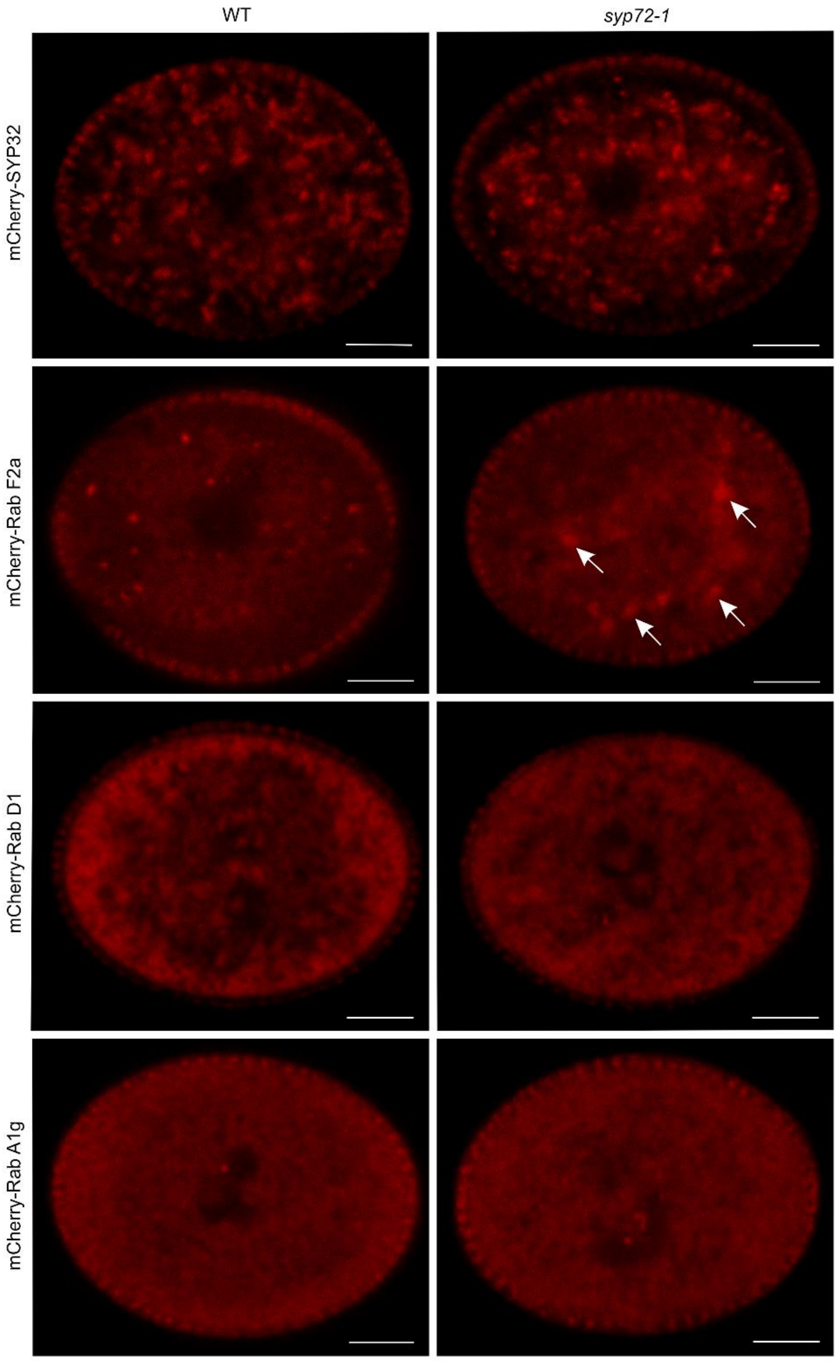

Supplementary Fig. 7 ***syp72*** **mutation leads to abnormal distribution of endosomes in pollen.**

Representative images of pollen from WT and *syp72-1* plants expressing *mCherry-SYP32* (Golgi apparatus), *mCherry-Rab F2a* (late endosomes), *mCherry-Rab D1* (post-Golgi) and *mCherry*-Rab A1g (recycling endosomes). (*n* = 13 biologically independent pollen grains for *mCherry-SYP32*; *n* = 10 for *mCherry-Rab F2a*, *mCherry-Rab D1* and *mCherry*-Rab A1g. Arrows indicate abnormal accumulation of *mCherry-Rab F2a*-labelled late endosomes. Scale bars, 5 μm.

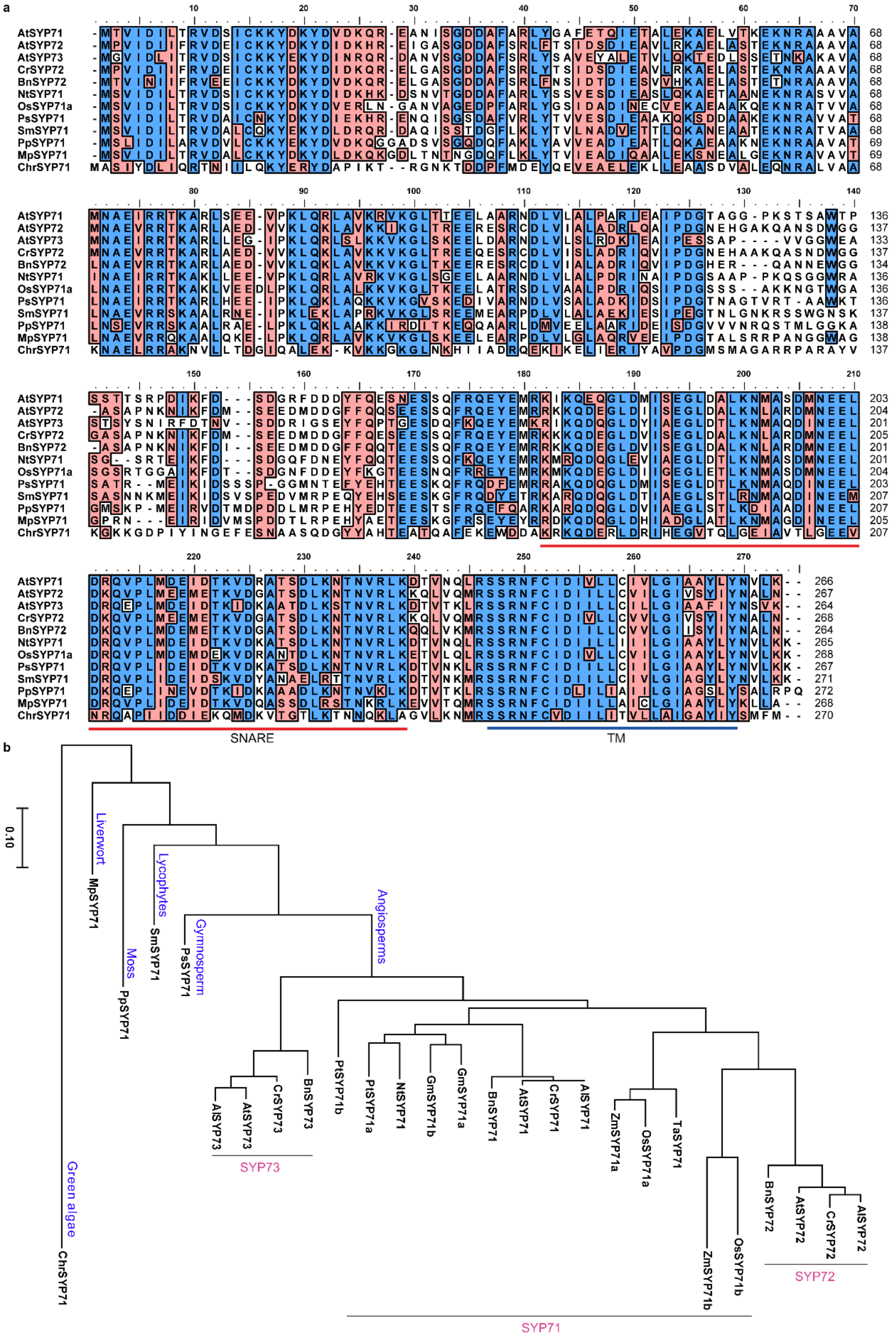

Supplementary Fig. 8 Sequence alignment and phylogenetic tree of SYP7 proteins from different species.

a Alignment of SYP7 protein sequences from different plant species. SNARE and transmembrane (TM) domains are outlined by red line and blue line, respectively. **b** Phylogenetic analysis of SYP7 proteins in the green lineage. The tree was constructed using the Maximum Likelihood method in MEGA7. *At*, *Arabidopsis thaliana*; *Al*, *Arabidopsis lyrata*; *Cr*, *Capsella rubella*; *Bn*, *Brassica napus*; *Nt*, *Nicotiana tabacum*; *Gm*, *Glycine max*; *Pt*, *Populus trichocarpa*; *Zm*, *Zea mays*; *Ta*, *Triticum aestivum*; *Os*, *Oryza sativa*; *Ps*, *Picea sitchensis*; *Sm*, *Selaginella moellendorffii*; *Pp*, *Physcomitrium (Physcomitrella) patens*; *Mp*, *Marchantia polymorpha*; *Chr*, *Chlamydomonas reinhardtii*.

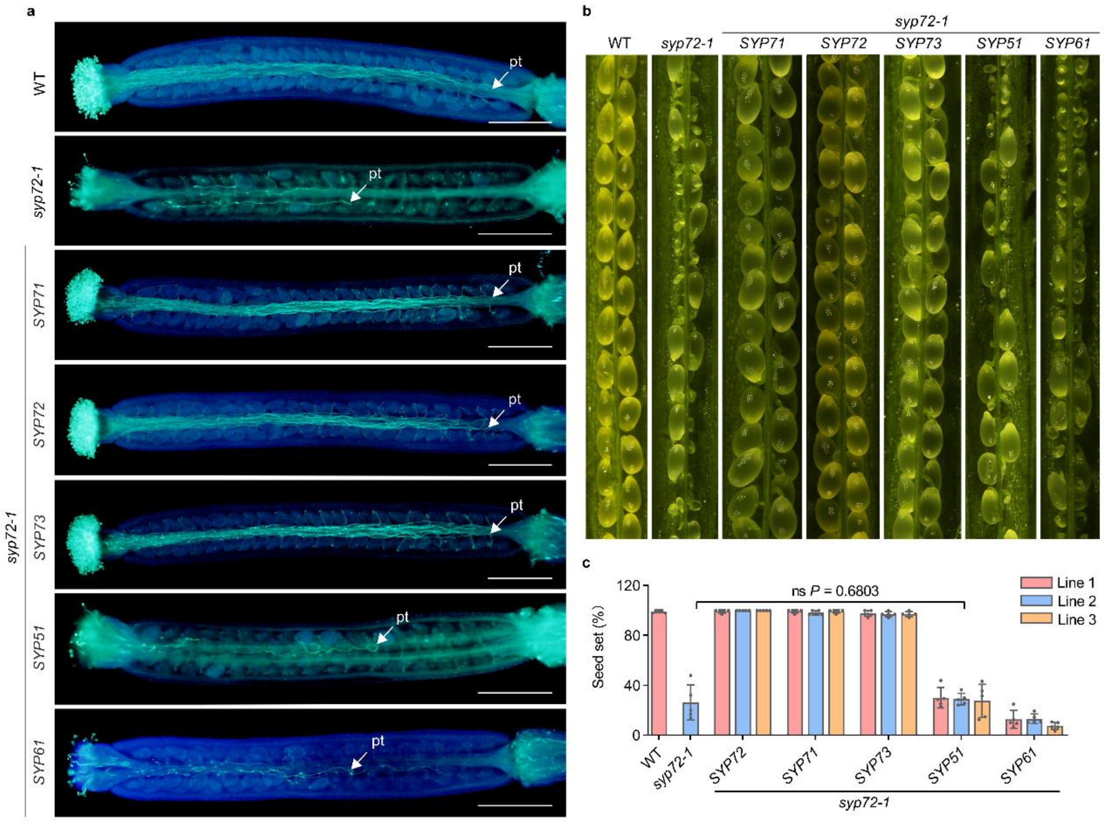

Supplementary Fig. 9 ***Arabidopsis SYP7*****, but not** ***SYP5*** **and** ***SYP6*** **family syntaxins could rescue the defects in pollen germination and seed setting of** ***syp72*** **mutant.**

a Representative images showing pollen tube growth of WT, s*yp72-1* and *syp72-1* mutant expressing *Arabidopsis SYP51*, *SYP61* and three *SYP7* family genes in the WT pistils at 24 HAP (*n* = 10 biologically independent pistils). Scale bars, 500 µm. **b** Representative images of siliques from the WT, *syp72-1* and *syp72-1* mutant expressing *Arabidopsis SYP51*, *SYP61* and three *SYP7* family genes. **c** Quantification of seed setting from WT, *syp72-1* and *syp72-1* mutant expressing *Arabidopsis SYP51*, *SYP61* and three *SYP7* family genes. Data represent the mean ± SD from five independent quantifications, with five siliques for each statistic (*n* = 25). Two-tailed Student's *t*-test was used for statistical analysis (ns, no significant difference, $P > 0.05$).

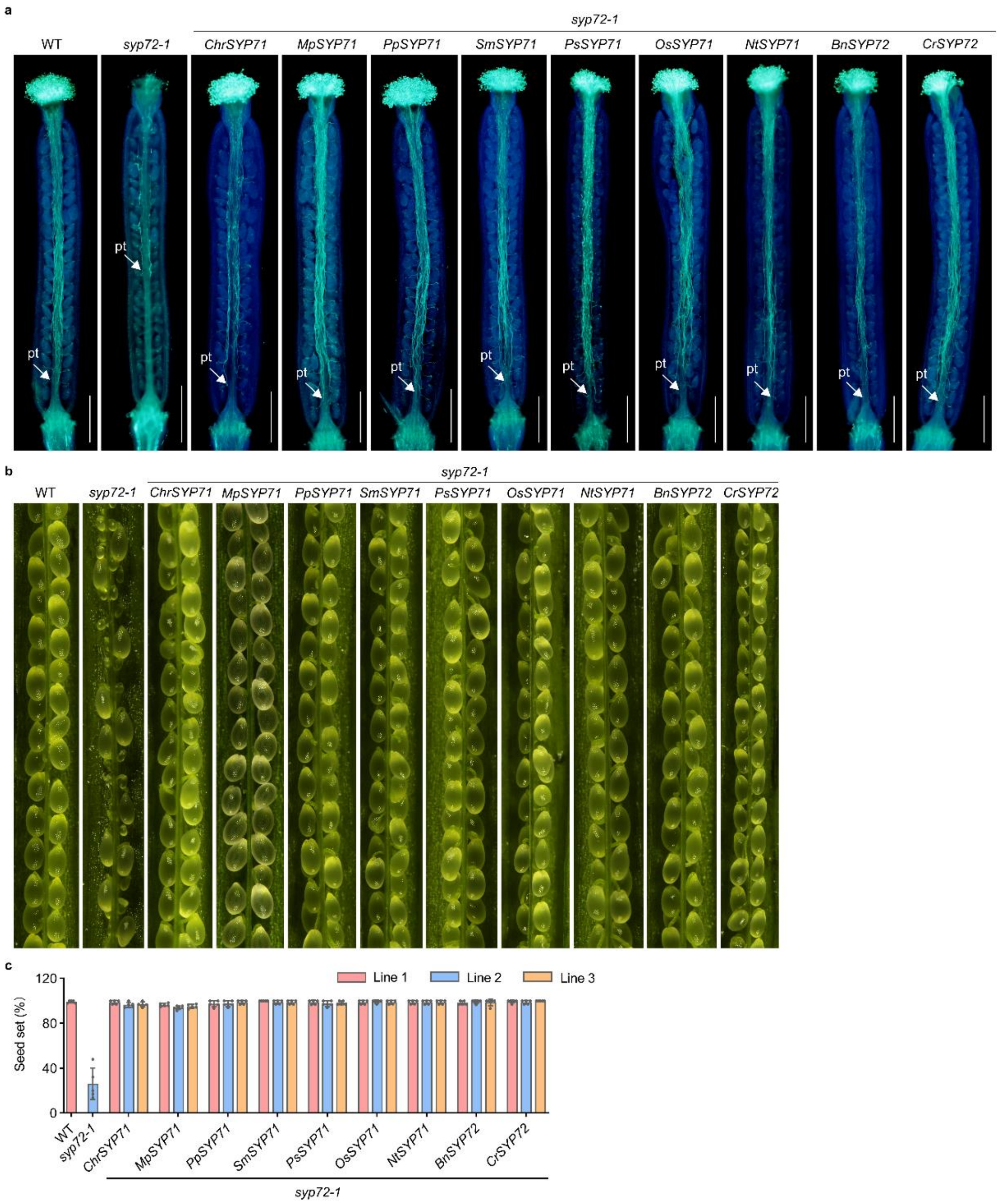

Supplementary Fig. 10 *SYP7* family syntaxins from green algae to angiosperms exhibit conserved molecular functions.

a Representative images showing pollen tube growth of WT, *syp72-1* and *syp72-1* mutant expressing *SYP7* family genes from green algae to angiosperms in the WT pistils at 24 HAP (*n* = 10 biologically independent pistils). Scale bars, 500 μm. **b** Representative images of siliques from WT, *syp72-1* and *syp72-1* expressing *SYP7* family genes from green algae to angiosperms. **c** Quantification of seed setting from WT, *syp72-1* and *syp72-1* expressing *SYP7* genes from green algae to angiosperms. Data represent the mean ± SD from five independent quantifications, with five siliques for each statistic (*n* = 25). *Chr*, *Chlamydomonas reinhardtii*; *Mp*, *Marchantia polymorpha*; *Pp*, *Physcomitrium* (*Physcomitrella*) *patens*; *Sm*, *Selaginella moellendorffii*; *Ps*, *Picea sitchensis*; *Os*, *Oryza sativa*; *Nt*, *Nicotiana tabacum*; *Bn*, *Brassica napus*; *Cr*, *Capsella rubella*.

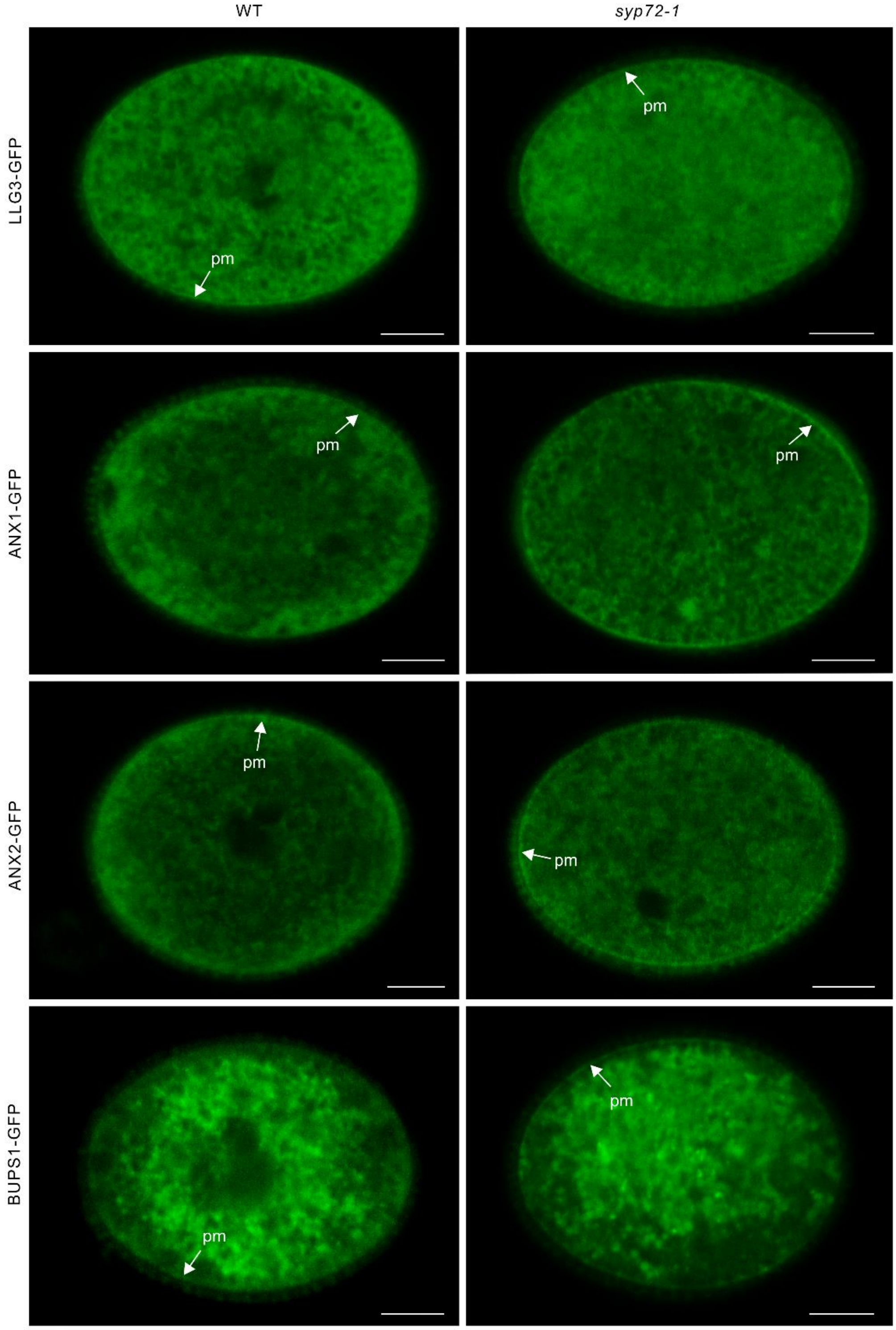

Supplementary Fig. 11 LLG3, ANX1/2 and BUPS1 could localize to the plasma membrane in *syp72-1* pollen.

Representative images showing LLG3-GFP, ANX1/2-GFP and BUPS1-GFP in WT and *syp72-1* pollen grains (n = 10 biologically independent pollen grains). WT, wild type; pm, plasma membrane. Scale bars, 5 µm.

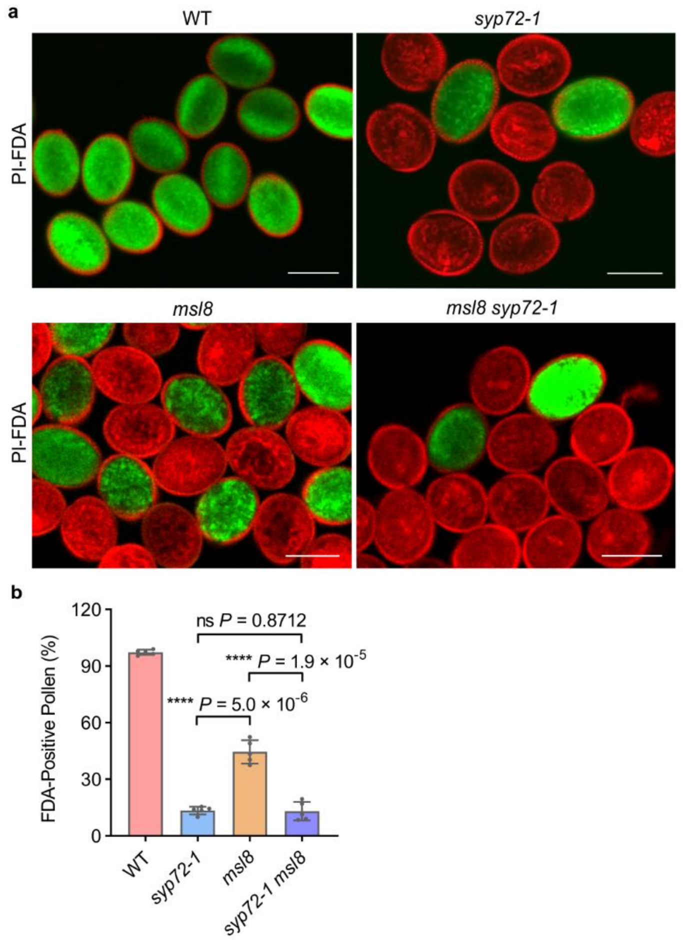

Supplementary Fig. 12 *msl8 syp72* double mutant displays the similar bursting phenotype as *syp72* single mutant during pollen rehydration.

a Representative images of Propidium iodide (PI) and fluorescein diacetate (FDA) double staining of mature pollen from the WT, *syp72-1*, *msl8* and *syp72-1 msl8* double mutants. Scale bars, 20 µm. **b** Percentages of FDA-positive pollen from the WT, *syp72-1*, *msl8* and *syp72-1 msl8* double mutants. Data represent the mean ± SD from five independent assays, with 200 pollen grains scored each time (n = 1,000). Two-tailed Student's t-test was used for statistical analysis (ns, no significant difference, $P > 0.05$; ****$P < 0.0001$)

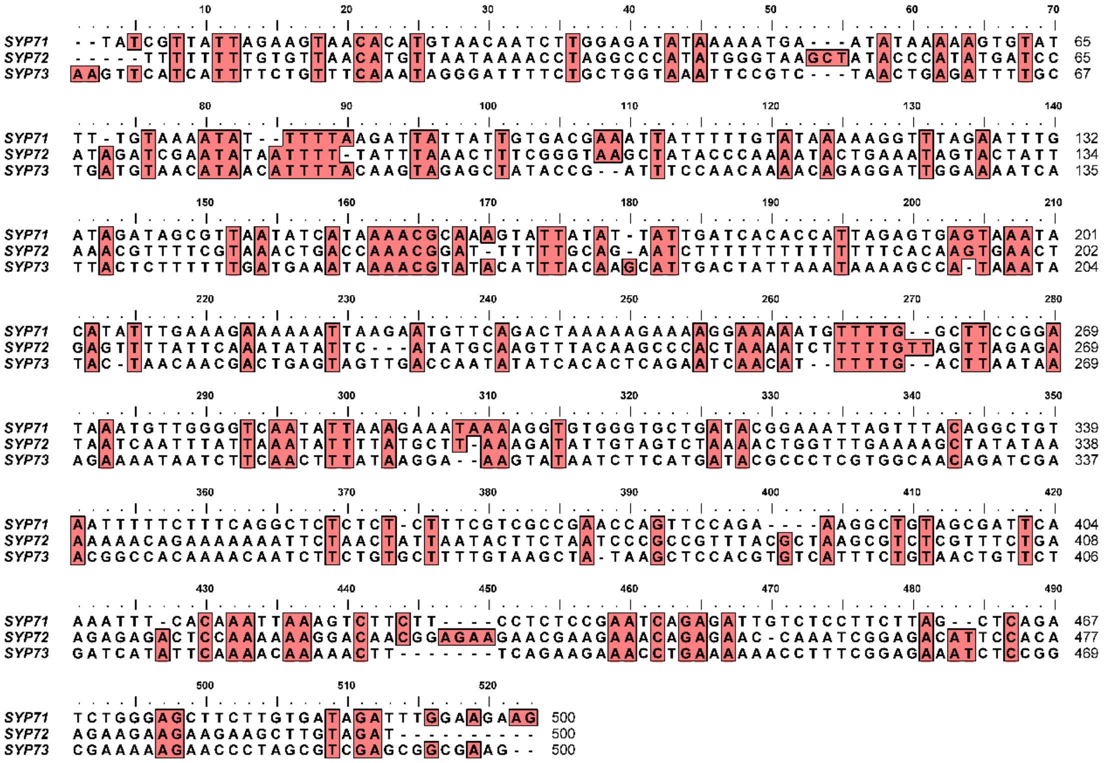

Supplementary Fig. 13 Sequence alignment of upstream 500 bp (of ATG) promoter sequences of *Arabidopsis SYP71*, *SYP72* and *SYP73*.

Conserved nucleotide sequences were outlined and shaded with pink color.

Supplementary Table 1. Transmission efficiency of *syp72* mutant alleles through male or female gamete

Parental Genotype		Progeny			Expected Ratio	χ2	*P* Value	TE^F	TE^M
Female	Male	Kan^r	Kan^s	Kan^r/Kan^s					
syp72-1/SYP72	WT	378	392	0.96	1:1	0.255	0.614	96.4%	N/A
WT	*syp72-1/SYP72*	12	756	0.02	1:1	720.75	9.2×10^{-159}	N/A	1.6%
syp72-3/SYP72	WT	486	494	0.98	1:1	0.065	0.798	98.4%	N/A
WT	*syp72-3/SYP72*	88	1240	0.07	1:1	999.33	2.5×10^{-219}	N/A	7.1%

TE^F and TE^M indicate transmission efficiency of female and male gamete respectively. N/A, not applicable.

Chi-square test (χ2) was performed for segregation ratios (1:1). One tailed *P* values were indicated in the Table.

Supplementary Table 2. Primers used in the present study

Name	Forward primer (5'-3')	Reverse primer (5'-3')	Experiment
SYP72	NNNGGTACCTTTATCTTGTGATCACTACTACGGCATTG	NNNGAATTCATCTACAAGCTTCTTCTTCTTCTTGTG	Promoter analysis
SYP72	NNNGGTACCTTTATCTTGTGATCACTACTACGGCATTG	NNNCTGCAGCCTAGTTTGAGAAATTGATAGCATTACC	Complementation
SYP72	NNNGCGGCCGCAATGCCGGTCATTGATATCATCTTCAG	NNNCCTAGGTTAGTTTAGAGCATTGTAAATGTAAG	CDS clone
SALK_005228	CGATAACTAGATCGCACCGAG	TTTCGTAAACTGACCAAACGG	Mutant identification
SALK_132295C	TTGCCTTTTTCGCAGATTATG	CTTTTTGCAAATCTCGTCGAC	Mutant identification
SALK_045561	CACAAAGCCAGAAGAATTTCG	AGAGAGGAACGAGAATCTCGG	Mutant identification
GK-579H09	GATCCTTTTATTCTCCGGTGCACC	AGTGGCTTAGCTTAGTCGCTATTA	Mutant identification
LB1.3	ATTTTGCCGATTTCGGAAC		Mutant identification
GK-LB	ATAATAACGCTGCGGACATCTACATTTT		Mutant identification
SYP71	NNNGCGGCCGCAATGACTGTGATCGATATTCTGACTAG	NNNCCTAGGGTTCCAGGACGAGGATCGCCAAGTGG	Gene clone
SYP73	NNNGCGGCCGCA ATGGGCGTAATTGATTTGATCACTAG	NNNCCTAGGGCTATAGATTTGCATTGGAGTTTAC	Gene clone
SYP51	GGCGAATTCCTCGAGGCGGCCGCAATGGCGTCTTCATCGGATTCATGG	TCCAAGCTTCCTAGGTGCGGCCGCTTACATATACTTAACCAACATCCATATTAC	CDS clone
SYP61	GGCGAATTCCTCGAGGCGGCCGCAATGTCTTCAGCTCAAGATCCATTCTAC	TCCAAGCTTCCTAGGTGCGGCCGCTTAGGTCAAGAAGACAAGAACGAATAGG	CDS clone
CrSYP72	GGCGAATTCCTCGAGGCGGCCGCA ATGCCTGTCATTGATATTATCTTTAG	TCCAAGCTTCCTAGGTGCGGCCGC TTACTTGTAGATGTAAGAAACGAT	CDS clone
BnSYP72	GGCGAATTCCTCGAGGCGGCCGCA ATGACGGTCATTAATATTATCTTCAG	TCCAAGCTTCCTAGGTGCGGCCGC TTAGTTCAGAGCATTATAGATGTAAG	CDS clone
NtSYP71	GGCGAATTCCTCGAGGCGGCCGCA ATGAGCGTAATAGATATACTGACAC	TCCAAGCTTCCTAGGTGCGGCCGC TTACTTCTTCAAGACATTGTACAAATAG	CDS clone
OsSYP71	GGCGAATTCCTCGAGGCGGCCGCA ATGAGCGTGATCGACATCCTCACGCGG	TCCAAGCTTCCTAGGTGCGGCCGCTCACTTTTTTAGAACATTGTAAAGATAAGCCG	CDS clone
PsSYP71	GGCGAATTCCTCGAGGCGGCCGCA ATGAGCGTGATCGACATTCTCACGC	TCCAAGCTTCCTAGGTGCGGCCGCTTATTTCTTCAAGACATTGTATAAGTAAG	CDS clone
SmSYP71	GGCGAATTCCTCGAGGCGGCCGCA ATGAGCGTCATCGACATTCTCACGC	TCCAAGCTTCCTAGGTGCGGCCGCTCATTTCTTCAAAACATTATACAAGTATC	CDS clone
PpSYP71	GGCGAATTCCTCGAGGCGGCCGCA ATGAGTTTAATCGACATTTTAGCTCG	TCCAAGCTTCCTAGGTGCGGCCGCTTATTGTGGTCGTAGTGCAGAGTATAG	CDS clone
MpSYP71	GGCGAATTCCTCGAGGCGGCCGCAATGAGTGTTATAGATATCTTGACTCG	TCCAAGCTTCCTAGGTGCGGCCGCTCAGGCCAATAATTTGTATAGATAGGCGG	CDS clone
ChrSYP71	GGCGAATTCCTCGAGGCGGCCGCA ATGGCTTCCATCTATGACCTTATCC	TCCAAGCTTCCTAGGTGCGGCCGCTTACATGAACATTGAATAGATGTAGGC	CDS clone
SYP72	NNNGGCCATTACGGCCATGCCGGTCATTGATATCATCTTCAG	NNNGGCCGAGGCGGCCGGTTTAGAGCATTGTAAATGTAAGAGAC	Yeast two-hybrid

MSL8	NNNGGCCATTACGGCCATGGATTTCAGAAATTCCTTCAAATCTC	NNNGGCCGCCTCGGCCTTGGCAGGTTGGTTTTGTGACCAGCC	Yeast two-hybrid
MSL9	CACACTAATCTAGACGGCCATTACGGCCATGGCTGAGAGGAGAGTCAGTAACGGAG	CCCATGGAGGCCTTTGGCCGAGGCGGCCTTTTTGTGACCAGTGAGATTGACATCTTG	Yeast two-hybrid
SYP72	ATATGCCGGTCATTGATATCATCTTC	GACAATTCCAAGGATCACACAGAG	RT-PCR
ACTIN 2	GAAGATTAAGGTCGTTGCACCACCTG	ATTAACATTGCAAAGAGTTTCAAGGT	RT-PCR
MSL8	GCAGGAGGAAGAGGTTGTGAG	CTGGAGCAATGTAATGGCGT	qPCR
AT1G58050	CCATTCTACTTTTTGGCGGCT	TCAATGGTAACTGATCCACTCTGATG	qPCR
AT4G34270	GTGAAAACTGTTGGAGAGAAGCAA	TCAACTGGATACCCTTTCGCA	qPCR
AT1G13320	TAACGTGGCCAAAATGATGC	GTTCTCCACAACCGCTTGGT	qPCR
RPL18aB	NNNGAGCTCTGGTGATCAAAGTCAATACTCATG	NNNTCTAGATTTTCGTCTGGAGAGAGACAATGC	Promoter clone
MSL8	NNN GCGGCCGC AATTCTGAAGCTATTTGGGTAAATACC	NNN CCTAGG GGCAGGTTGGTTTTGTGACCAGCCAG	Subcellular localization analysis
MSL8	NNN CCTAGG ATGGATTTCAGAAATTCCTTCAAATCTC	NNN GAATTC GGCAGGTTGGTTTTGTGACCAGCC	Co-IP
MBP-SYP72	NNN CATATG ATGGGAAGAGCCATGGATAAAATCG<br>GAAGCCCTGAAAGACGCGCAGACT CCGGTCATTGATATCATCTTCAG	CTGAAGATGATATCAATGACCGGAGTCTGCGCGTCTTTCAGGGCTTC<br>NNN CTCGAG GTTTAGAGCATTGTAAATGTAAGAGAC	Pull Down
MBP	NNN CATATG ATGGGAAGAGCCATGGATAAAATCG	NNN CTCGAG AGTCTGCGCGTCTTTCAGGGCTTCATC	Pull Down
Lat52	NNN GAGCTC TGTCGACATACTCGACTCAGAAGG	NNN CTCGAG TTTAAATTGGAATTTTTTTTTTTGGTGTGTG	Promoter clone
ST-RFP	NNN TCTAGA ATGATTCATACCAACTTGAAG<br>GAGAACTTGTTTCAAATGGAACCGTTACC GTGTCTAAGGGCGAAGAGC	CGGTTCCATTTGAAACAAGTTCTCCAGTCGCAGT CATGGCCACTTTCTCCTGGCTC<br>NNN AAGCTT TTACTTGTACAGCTCGTCCATGCC	ST-RFP marker
ANX1	CCAACGCGTTGG GAGCTC GGTACC GTGCCCAATGTCTCAACCTCTGCTTC	CTTCCTAGGTGCGGCCGC CTCGAG TCGTCCTTTGGGATTTACAATCTGAGAG	Subcellular localization analysis
ANX2	CCAACGCGTTGG GAGCTC GGTACC GGCTTTGCTTCAGGGTCTAAATGTATG	CTTCCTAGGTGCGGCCGC CTCGAG TCGTCCTTTAGGGTTTACAATCTGAG	Subcellular localization analysis
BUPS1	CCAACGCGTTGG GAGCTC GGTACC ATAGAAATTGGTGATTGAAGTTTGATTG	CTTCCTAGGTGCGGCCGC CTCGAG TCTTCCGTTAAGGTTAGCAAACTGAG	Subcellular localization analysis
