# Supplementary material for: SYP72 interacts with the mechanosensitive channel MSL8 to protect pollen from hypoosmotic shock during hydration
Source: Nat Commun. 2022 Jan 10;13:73. doi: 10.1038/s41467-021-27757-9 (PMC8748641; doi:10.1038/s41467-021-27757-9)
Supplement: Supplementary file 3 — Reporting Summary [file 41467_2021_27757_MOESM3_ESM.pdf]

nature portfolio

Corresponding author(s): Peng Zhao

Last updated by author(s): Nov 26, 2021

Reporting Summary

Nature Portfolio wishes to improve the reproducibility of the work that we publish. This form provides structure for consistency and transparency in reporting. For further information on Nature Portfolio policies, see our Editorial Policies and the Editorial Policy Checklist.

Statistics

For all statistical analyses, confirm that the following items are present in the figure legend, table legend, main text, or Methods section.

n/a	Confirmed	
☐	☒	The exact sample size (*n*) for each experimental group/condition, given as a discrete number and unit of measurement
☐	☒	A statement on whether measurements were taken from distinct samples or whether the same sample was measured repeatedly
☐	☒	The statistical test(s) used AND whether they are one- or two-sided<br>*Only common tests should be described solely by name; describe more complex techniques in the Methods section.*
☒	☐	A description of all covariates tested
☒	☐	A description of any assumptions or corrections, such as tests of normality and adjustment for multiple comparisons
☐	☒	A full description of the statistical parameters including central tendency (e.g. means) or other basic estimates (e.g. regression coefficient) AND variation (e.g. standard deviation) or associated estimates of uncertainty (e.g. confidence intervals)
☐	☒	For null hypothesis testing, the test statistic (e.g. *F*, *t*, *r*) with confidence intervals, effect sizes, degrees of freedom and *P* value noted<br>*Give P values as exact values whenever suitable.*
☒	☐	For Bayesian analysis, information on the choice of priors and Markov chain Monte Carlo settings
☒	☐	For hierarchical and complex designs, identification of the appropriate level for tests and full reporting of outcomes
☐	☒	Estimates of effect sizes (e.g. Cohen's *d*, Pearson's *r*), indicating how they were calculated

Our web collection on statistics for biologists contains articles on many of the points above.

Software and code

Policy information about availability of computer code

Data collection	Microscopic images were acquired with the Leica LAS X (ver. 3.5.5.19976). RT-qPCR data were collected using the Bio-Rad CFX Manager (ver. 3.1). TEM images were collected with the Gatan Microscopy Suite (GMS) 3.
Data analysis	ImageJ (ver.1.52a) was used for image analysis. GraphPad Prism (v.8.4.2) was used to prepare line, bar and box-and-whisker plots. The fluorescence intensities of pollen were measured using LAS-X (ver. 3.5.5.19976). IBM SPSS Statistics 23 and GraphPad Prism (v.8.4.2) were used for statistical analyses. MEGA 7 was used for phylogenetic analysis. Bowtie 2 and RSEM were used for RNA-data analysis. RT-qPCR data were analyszed using the Bio-Rad CFX Manager (ver. 3.1).

For manuscripts utilizing custom algorithms or software that are central to the research but not yet described in published literature, software must be made available to editors and reviewers. We strongly encourage code deposition in a community repository (e.g. GitHub). See the Nature Portfolio guidelines for submitting code & software for further information.

Data

Policy information about availability of data

All manuscripts must include a data availability statement. This statement should provide the following information, where applicable:

- Accession codes, unique identifiers, or web links for publicly available datasets
- A description of any restrictions on data availability
- For clinical datasets or third party data, please ensure that the statement adheres to our policy

Publicly-available RNA-seq datasets including seedlings (GSE32318), stems (GSE102694), roots and rosettes (GSE87760), carpel (GSE56326),floral bud (GSE45685), ovule (DRR044369), mature pollen (PRJNA194429), pollen tube (GSE98145) were used for expression level analysis. All data are available from the corresponding author upon request.

Field-specific reporting

Please select the one below that is the best fit for your research. If you are not sure, read the appropriate sections before making your selection.

☒ Life sciences ☐ Behavioural & social sciences ☐ Ecological, evolutionary & environmental sciences

For a reference copy of the document with all sections, see nature.com/documents/nr-reporting-summary-flat.pdf

Life sciences study design

All studies must disclose on these points even when the disclosure is negative.

Sample size	Sample sizes were estimated based on our experiences in performing similar experiments and published published studies. Sample sizes are available for each figure and individual data points are displayed.
Data exclusions	No data exclusion.
Replication	At least three biological replicates were performed from all biological experiments.
Randomization	Typical randomization was not necessary for this type of experiment, although all plant materials with same genotype were randomly picked.
Blinding	This experiment is not of typical blinding type although all phenotype analysis was performed randomly among different genotypes.

Reporting for specific materials, systems and methods

We require information from authors about some types of materials, experimental systems and methods used in many studies. Here, indicate whether each material, system or method listed is relevant to your study. If you are not sure if a list item applies to your research, read the appropriate section before selecting a response.

Materials & experimental systems

n/a	Involved in the study	
☐	☒	Antibodies
☒	☐	Eukaryotic cell lines
☒	☐	Palaeontology and archaeology
☒	☐	Animals and other organisms
☒	☐	Human research participants
☒	☐	Clinical data
☒	☐	Dual use research of concern

Methods

n/a	Involved in the study	
☒	☐	ChIP-seq
☒	☐	Flow cytometry
☒	☐	MRI-based neuroimaging

Antibodies

Antibodies used	Mouse anti GFP (Abclonal; Catalog number: AE012; 1:1,000 dilution)<br>Mouse anti MYC (Abclonal; Catalog number: AE010; 1:1,000 dilution)<br>Mouse anti MBP (NEB; Catalog number: E8032S; 1:1,000 dilution)<br>Plant Actin Mouse Monoclonal Antibody (Abbkine; Catalog number: A01050; 1:1,000 dilution)
Validation	GFP antibody was bought from Abclonal (https://www.abclonal.com.cn/catalog/AE012).<br>MYC antibody was bought from Abclonal (https://abclonal.com.cn/catalog/AE010).<br>MBP antibody was bought from NEB (http://www.neb-china.com/pshow.asp?id=3612).<br>Plant Actin Mouse Monoclonal Antibody was bought from Abbkine (https://www.abbkine.com/product/anti-plant-actin-mouse-monoclonal-antibody-3t3-a01050/)
